# Short- and long-term outcomes of minimally invasive total mesorectal excision in obese versus nonobese patients with rectal cancer: a propensity score matched study

**DOI:** 10.1007/s10151-026-03292-x

**Published:** 2026-05-12

**Authors:** G.-Y. Chen, K.-Y. Tsai, C.-K. Liao, J.-F. You, C.-C. Lai, S.-H. Huang

**Affiliations:** 1 Department of Surgery, Chang Gung Memorial Hospital, Linkou, No. 5, Fuxing St., Guishan Dist., Taoyuan, 333 Taiwan; 2 Division of Colon and Rectal Surgery, Department of Surgery, Chang Gung Memorial Hospital, Linkou, No. 5, Fuxing St., Guishan Dist., Taoyuan, 333 Taiwan; 3 School of Medicine, Chang Gung University, No. 259, Wenhua 1st Road, Guishan Dist., Taoyuan, 333 Taiwan; 4 Division of Colon and Rectal Surgery, New Taipei Municipal TuCheng Hospital, No. 6, Sec. 2, Jincheng Rd., Tucheng Dist, New Taipei City, 236043 Taiwan, ROC

**Keywords:** Obesity, Rectal cancer, Total mesorectal excision, Minimally invasive surgery, Retrospective study

## Abstract

**Background:**

Obesity adds technical complexity to colorectal surgery and has been linked to higher rates of perioperative complications and poorer long-term outcomes. Its prevalence is increasing among patients undergoing minimally invasive total mesorectal excision for rectal cancer; however, its impact on perioperative and oncologic outcomes remains controversial. Therefore, the aim of this study was to compare short- and long-term outcomes between obese (body mass index (BMI) ≥ 27 kg/m^2^) and nonobese patients undergoing minimally invasive total mesorectal excision.

**Methods:**

Retrospective review of patients with rectal cancer undergoing laparoscopic, robotic, or transanal total mesorectal excision (TME) between January 2015 and December 2022. Propensity score matching (1:1) was performed on baseline characteristics. Primary outcomes included perioperative parameters, postoperative complications, and long-term oncologic outcomes.

**Results:**

After matching, 142 patients were included in each group. Obesity was associated with higher conversion rates to open surgery (2.8% versus 0%, *p = *0.044) and increased overall complications (40.1% versus 28.9%, *p = *0.046), driven mainly by surgical wound infections (9.2% versus 1.4%, *p = *0.004). No differences were observed in major complications, anastomotic leakage, hospital stay, margin status, or 5-year overall (88% versus 89.4%, *p = *0.409) and disease-free (62.7% versus 72.5%, *p = *0.653) survival.

**Conclusions:**

Obesity increased conversion and minor complication rates but did not adversely affect short-term outcomes or long-term oncologic outcomes after minimally invasive TME procedures. Tailored perioperative strategies may mitigate obesity-associated risks.

**Trial registration:**

Not applicable.

**Supplementary Information:**

The online version contains supplementary material available at 10.1007/s10151-026-03292-x.

## Introduction

Colorectal cancer (CRC) remains a leading cause of cancer-related morbidity and mortality worldwide, with rectal cancer accounting for approximately one-third of all cases [1]. Surgical resection remains key to curative outcomes regarding the management of locally advanced rectal cancer (LARC). Total mesorectal excision (TME) has revolutionized the surgical management of LARC, significantly reducing local recurrence rates and improving long-term survival by ensuring complete removal of the mesorectal envelope and achieving negative circumferential resection margins. In recent years, the three minimally invasive TME techniques, laparoscopic TME (LapTME), robotic TME, and transanal TME (TaTME), have demonstrated noninferior oncologic outcomes and improved short-term outcomes such as shorter hospital stays, fewer postoperative complications, and faster functional recovery compared with conventional open TME [2, 3]. With continuing advancements in surgical instrumentation and techniques, minimally invasive surgery (MIS) has emerged as the standard treatment for CRC.

In many Asian countries, obesity is often defined as body mass index (BMI) exceeding 27 kg/m^2^, whereas Western populations typically use a threshold of 30 kg/m^2^, owing to differences in body fat distribution [4]. The global prevalence of obesity has demonstrated a steadily increasing trajectory. Data from many Asian countries have indicated a steady increase in the prevalence of overweight and obesity, paralleling economic growth and concurrent changes in dietary habits [5]. The adoption of Westernized lifestyles may contribute to the rising prevalence of obesity observed in Asian countries. There is a general consensus that CRC serves as a marker of socioeconomic development, with incidence rates closely reflecting the human development index [6]. Consequently, obesity is increasingly prevalent among patients presenting for rectal cancer surgery. Obesity is associated with numerous adverse outcomes, including metabolic, cardiovascular, musculoskeletal, neurological, respiratory, and gastrointestinal complications, all of which are generally considered to have a greater risk of perioperative complications and technical difficulties compared with normal-weight patients [7]. The technical complexity of TME is significantly heightened in obese patients due to increased visceral adiposity, bulky mesentery, narrow pelvic anatomy, and distorted tissue planes, all of which are associated with increased intraoperative difficulty in MIS and may collectively contribute to prolonged operative times, increased blood loss, and elevated conversion to open rates, reported by meta-analyses and large retrospective studies [8–10]. Moreover, thickened abdominal wall can make ostomy creation more challenging.

While many surgeons acknowledge the technical challenges of performing TME in obese patients, considerable disagreement exists in the literature regarding the impact of obesity on surgical outcomes. Several large-scale retrospective and prospective studies have demonstrated that while obese patients experience longer operative times and extended hospital stays, their postoperative complication rates, positive circumferential resection margin rates, lymph node harvest yields, and long-term oncologic outcomes are comparable to those of nonobesity patients when treated at specialized centers [10, 11]. Further, other studies demonstrated increased rates of wound and cardiopulmonary complications and even anastomotic leakage rates in obese patients [9, 12]. Given the high prevalence of obesity and the technical challenges inherent in rectal cancer surgery, elucidating the impact of obesity on this patient population is essential for optimizing individualized surgical planning. To address these crucial issue, this study aimed to compare perioperative, short-, and long-term outcomes between obese (BMI ≥ 27 kg/m^2^) and nonobese patients undergoing minimally invasive TME at a high-volume tertiary referral center.

## Materials and methods

### Patient selection

This retrospective study reviewed patients who underwent restorative proctectomy for rectal cancer at Chang Gung Memorial Hospital between January 2015 and December 2022. The study was approved by the Institutional Review Board (approval no.: 202501451B0), and the requirement for informed consent was waived due to the use of de-identified data. This research received no external funding. Due to the retrospective design of the study, the local ethics committee confirmed that informed consent from participants was not necessary.

**Fig. 1:**
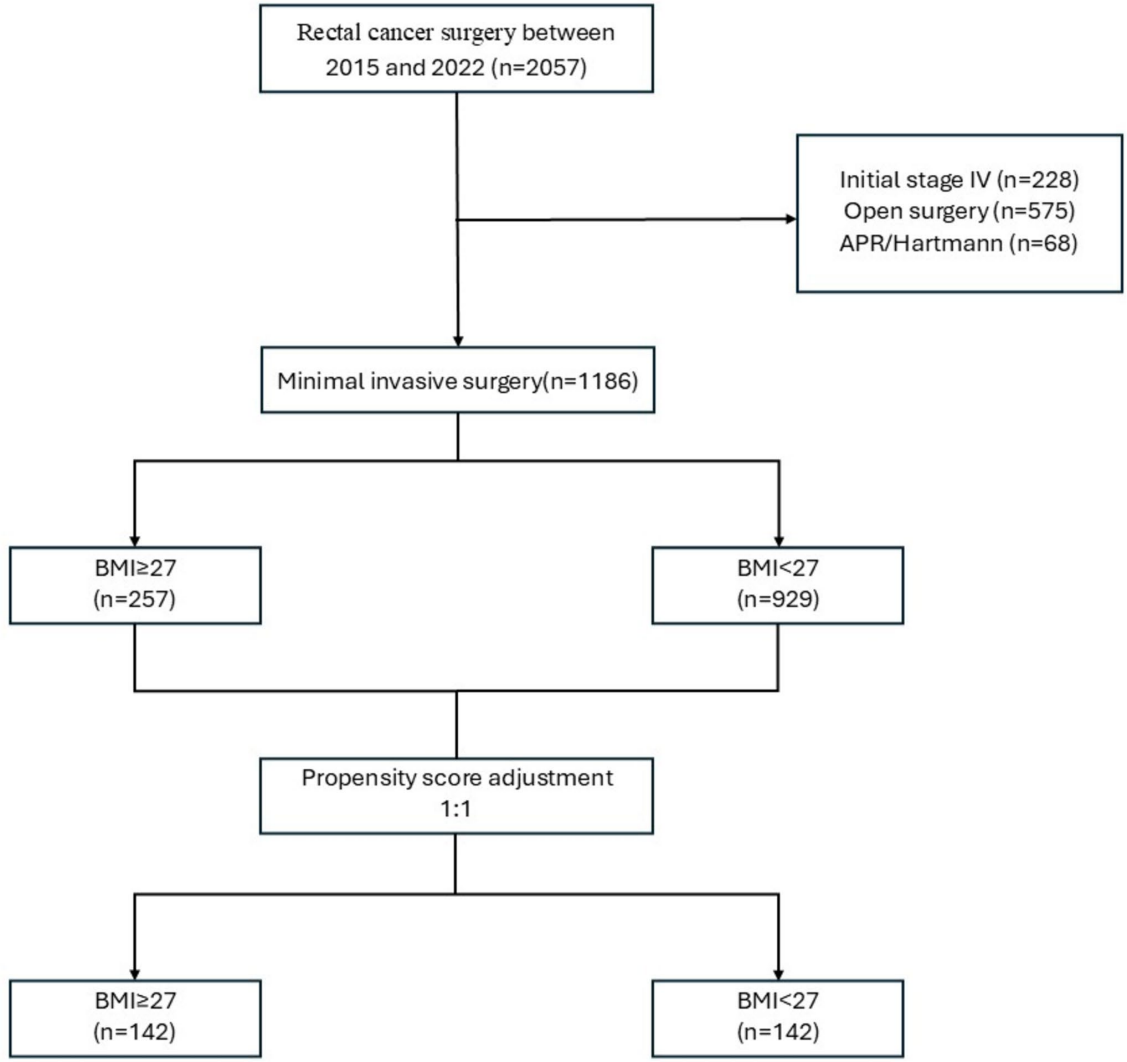
Selection process of patient data

Eligible participants were identified from patients with rectal cancer who had undergone comprehensive diagnostic evaluation, including colonoscopy with tissue biopsy, chest–pelvic computed tomography (CT), rectal magnetic resonance imaging (MRI), and endorectal ultrasonography when clinically indicated. Specific inclusion and exclusion criteria were established to define the study population. Inclusion criteria comprised patients with histologically confirmed rectal adenocarcinoma located within 15 cm from the anal verge who were considered candidates for TME with primary anastomosis. Tumor location was determined using rigid proctoscopy to measure the distance from the anal verge. Exclusion criteria included patients requiring conventional open surgery, patients presenting with stage IV disease at initial diagnosis, and those undergoing alternative surgical approaches such as abdominoperineal resection (APR) or Hartmann’s procedure. The detailed selection process is illustrated in Fig. 1.

### Assessment and treatment protocol

Prior to surgical intervention, all patients underwent standardized preoperative workup consisting of thorough physical assessment, digital rectal examination, complete colonoscopic evaluation with tissue biopsy, and chest–pelvis CT. MRI of the pelvis was performed to characterize local tumor extent and regional involvement patterns. Carcinoembryonic antigen (CEA) were obtained as part of standard laboratory evaluation.

Pathological staging was determined using the 8th edition of the Union for International Cancer Control (UICC) tumor node metastasis (TNM) classification system [13]. Treatment planning decisions were formulated through multidisciplinary team consultation, incorporating input from surgical, medical, and radiation oncology specialists. The implementation of neoadjuvant therapy protocols was individualized on the basis of staging results and multidisciplinary consensus recommendations.

The selection between surgical techniques was determined through consideration of surgeon expertise, tumor features, and patient-specific clinical factors. The construction of protective stoma was performed on the basis of intraoperative surgical assessment and surgeon clinical judgment.

### Data collection

Clinical information was systematically retrieved from our institution’s electronic medical records. Baseline patient characteristics included demographic data, clinical parameters, laboratory results (serum albumin and CEA levels), tumor characteristics (location and TNM stage), the use of neoadjuvant and adjuvant therapies, and previous abdominal surgery (appendectomy, cholecystectomy, gastrectomy, hysterectomy, oophorectomy, and colon operation).

The analysis covered clinical and procedural factors such as surgical details (blood loss, stoma creation, conversion to open surgery) and anastomotic techniques. Pathological evaluation included tumor histology, differentiation, lymphovascular and perineural invasion, circumferential and distal resection margins (CRM and DRM, respectively), and lymph node yield. Margins were considered positive if tumor cells were present within 1 mm of the specimen edge.

Perioperative morbidity was stratified according to the Clavien–Dindo classification system, with major complications categorized as grade III or above [14]. Mortality rates were documented throughout the study period. Long-term oncological endpoints included overall survival (OS), disease-free survival (DFS), local recurrence (LR), and distant metastasis (DM).

### Surgical techniques

All minimally invasive TME procedures in this cohort were performed according to standard oncologic surgical principles. High ligation of the inferior mesenteric artery (IMA) or low ligation with IMA root lymph node dissection was routinely carried out, depending on the surgeon’s professional judgment. Mobilization of the splenic flexure was performed selectively when needed to construct a tension-free colorectal or coloanal anastomosis. Anastomotic integrity was assessed intraoperatively using a routine air-leak test when clinically deemed appropriate, typically via endoscopic visualization or air-insufflation submersion techniques. Operations were performed by expertise colorectal surgeons, all trained at tertiary referral centers with substantial experience in LapTME, robotic TME, and TaTME. The surgical approach for each case was determined by the operating surgeon on the basis of their expertise and case-specific clinical complexity. Procedures were conducted by different surgeons working independently.

### Follow-up

Postoperative monitoring adhered to our department’s standardized protocol. Clinical assessments occurred at 3–6-month intervals during the initial 3-year period, followed by annual evaluations. Each consultation included comprehensive physical examination, CEA determination, and appropriate imaging studies for recurrence detection.

The 5-year follow-up strategy incorporated annual whole-body CT and endoscopic evaluation of the colorectum. Accelerated diagnostic workup was initiated when clinical presentation warranted earlier assessment for suspected disease recurrence.

OS was calculated from the operative date to patient death or most recent clinical contact. DFS was measured from surgery to documented recurrence, death, or final follow-up assessment. Data collection continued through 7 August 2025 to support adequate duration for survival endpoint analysis.

### Statistical analysis

Quantitative variables were presented as medians with interquartile ranges (IQR) and analyzed using the Mann–Whitney *U* nonparametric test for between-group comparisons. Categorical variables were summarized through frequencies and percentages, analyzed via chi-squared or Fisher’s exact test as appropriate. Covariate balance before and after matching was assessed using both *p*-values and standardized mean differences (SMDs). An absolute SMD < 0.1 was considered to reflect negligible imbalance, whereas SMD values between 0.1 and 0.2 were interpreted as indicating small imbalance. Survival outcomes were evaluated through Kaplan–Meier survival curves, with between-group differences assessed using log-rank testing. Univariate logistic regression was used to identify risk factors for postoperative complications.

Propensity scores were calculated using logistic regression on the basis of key preoperative variables, including tumor location, serum albumin level, American Society of Anesthesiology Score (ASA) score, and the use of neoadjuvant chemotherapy, and oxaliplatin frequency. Variables with significant baseline differences among groups informed the matching criteria. A 1:1 propensity score matching (PSM) was performed between the obesity and nonobesity group to establish comparable baseline characteristics and minimize selection bias. Statistical significance was set at *p* < 0.05. All analyses were performed using SPSS version 26 (IBM Corp., New York, NY, USA).

## Results

### Patient characteristics before and after PSM adjustment

Baseline and preoperative clinicopathological parameters are presented in Table 1, demonstrating comparisons before and after PSM. A total of 1186 patients were included prior to matching: 257 in the BMI ≥ 27 kg/m^2^ group and 929 in the BMI < 27 kg/m^2^ group. Prior to matching, the cohorts exhibited statistically significant differences across several key variables, including tumor location (*p* = 0.045), serum albumin level (*p* = 0.028), ASA score (*p* < 0.001), and use of neoadjuvant chemotherapy (*p* = 0.021) and oxaliplatin-containing regimens (*p* = 0.011).

**Table 1:** Basic characteristics of obese and nonobese groups with rectal cancer who underwent restorative proctectomy before and after propensity score matching

	Before propensity score matching			SMD	After propensity score matching		*p*-Value	SMD
	BMI ≥ 27 (*n* = 257)	BMI < 27 (*n* = 929)	*p*-Value		BMI ≥ 27 (*n* = 142)	BMI < 27 (*n* = 142)		
Age	61 (14)	63 (16)	0.097	−0.174	60 (13)	64 (16)	0.065	−0.032
Gender								
Male	169 (65.8)	572 (61.6)	0.220	0.087	100 (70.4)	86 (60.6)	0.081	0.207
Female	88 (34.2)	357 (38.4)		−0.087	42 (29.6)	56 (39.4)		− 0.207
BMI	29.0 (2.6)	23.3 (3.7)	< 0.001*	2.542	28.7 (2.4)	23.3 (3.6)	< 0.001*	2.671
Surgical type								
TaTME	42 (16.3)	113(12.2)	0.095	0.120	40 (28.2)	47 (33.1)	0.271	− 0.107
Robotic LAR	12 (4.7)	67(7.2)		−0.108	15 (10.6)	21 (14.8)		−0.117
LapTME	203 (79)	749(80.6)		−0.041	87 (61.3)	74 (52.1)		0.185
Tumor location								
Upper rectum	74 (28.8)	250(26.9)	0.045*	0.042	15 (10.6)	24 (16.9)	0.121	−0.184
Middle rectum	124 (48.2)	520 (56)		−0.155	68 (47.9)	53 (37.3)		0.214
Lower rectum	59 (23)	159(17.1)		0.144	59 (41.5)	65 (45.8)		−0.085
Albumin								
< 3.5	6 (2.3)	53 (5.7)	0.028*	−0.172	4 (2.8)	2 (1.4)	0.409	0.098
≥ 3.5	251 (97.7)	876 (94.3)		0.172	138 (97.2)	140 (98.6)		−0.098
CEA								
< 5	199 (77.4)	705 (75.9)	0.607	0.036	114 (80.3)	113 (79.6)	0.882	0.034
≥ 5	58 (22.6)	224 (24.1)		−0.036	28 (19.7)	29 (20.4)		−0.034
ASA score								
2	83 (32.3)	450 (48.4)	< 0.001*	0.329	67 (47.2)	68 (47.9)	0.905	0.014
3	174 (67.7)	479 (51.6)		0.329	75 (52.8)	74 (52.1)		−0.014
pT-stage								
T0	15 (5.8)	35 (3.8)	0.098	0.097	13 (9.2)	7 (4.9)	0.305	0.165
T1	51 (19.8)	134 (14.4)		0.144	21 (14.8)	16 (11.3)		0.105
T2	45 (17.5)	199 (21.4)		−0.100	30 (21.1)	36 (25.4)		−0.119
T3	126 (49)	479 (51.6)		−0.051	71 (50)	70 (49.3)		0.034
T4	20 (7.8)	82 (8.8)		−0.035	7 (4.9)	13 (9.2)		0.244
pN-stage								
N0	151 (58.8)	555 (59.7)	0.465	−0.015	91 (64.1)	86 (60.6)	0.623	0.073
N1	66 (25.7)	256 (27.6)		0.054	38 (26.8)	38 (26.8)		0.000
N2	40 (15.6)	118 (12.7)		−0.037	13 (9.2)	18 (12.7)		−0.103
Tumor size	4.4 (6)	4.6 (7.2)	0.657	−0.035	4.8 (6.3)	5.1 (6.5)	0.061	−0.048
Neoadjuvant treatment								
Yes	150 (58.4)	534 (57.5)	0.799	0.018	94 (66.2)	99 (69.7)	0.525	−0.075
No	107 (41.6)	395 (42.5)		−0.018	48 (33.8)	43 (30.3)		0.075
Neoadjuvant RT	74 (28.8)	272 (29.3)	0.880	−0.011	59 (41.5)	68 (47.9)	0.283	−0.107
Neoadjuvant chemotherapy	88 (34.2)	250 (26.9)	0.021*	0.159	75 (52.8)	82 (57.7)	0.403	−0.099
Adjuvant chemotherapy	126 (49)	450 (48.4)	0.867	0.012	80 (56.3)	80 (56.3)	1	0.000
Chemotherapy with oxaliplatin	52 (20.2)	261 (28.1)	0.011*	−0.184	37 (26.1)	40 (28.2)	0.689	−0.048
Previous abdominal surgery								
Yes	90 (35.0)	311 (33.5)	0.754	0.033	46 (32.4)	50 (35.2)	0.684	−0.059
No	167 (65.0)	618 (66.5)			96 (67.6)	92 (64.8)		0.059

*BMI* body mass index, *SMD* standardized mean difference, *LapTME* laparoscopic total mesorectal excision, *TaTME* transanal total mesorectal excision, *AV* anal verge, *ASA score* American Society of Anesthesiology Score

After propensity score matching, 142 patients were included in each group. Matching achieved good balance across most baseline variables, eliminating statistically significant differences in tumor location, serum albumin level, ASA score, and the use of neoadjuvant chemotherapy or oxaliplatin-containing regimens. Absolute SMDs were < 0.1 for the majority of covariates, indicating adequate balance. To further examine the robustness of our matching approach, we constructed an alternative propensity score model that additionally incorporated age, gender, and all variables demonstrating either *p* < 0.05 or |SMD| > 0.1 before matching. Although this expanded model improved balance across several covariates, it substantially reduced the number of matched pairs from 142 versus 142 to 41 versus 41 without materially altering the effect estimates. Given the considerable loss of sample size and consequent reduction in statistical power, the original matched cohort was retained as the primary analytic dataset. Residual imbalances in gender was addressed through multivariable adjustment and stratified analyses, as presented in Supplementary Table S1.

### Operative parameters and short-term outcomes

Operative parameters are summarized in Table 2. After PSM, operative time showed no significant difference between the groups (312 ± 139 min versus 280 ± 118 min, *p* = 0.287). Estimated blood loss was similar between the two groups (50 ± 55 ml versus 50 ± 30 ml, *p* = 0.070). The rate of diverting stoma creation did not differ significantly (50.7% versus 45.1%, *p* = 0.342). Conversion to open surgery occurred only in the obesity group (2.8% versus 0%, *p* = 0.044); three conversions were due to adhesions and one to advanced disease. Anastomosis methods were comparable between the two groups (*p* = 0.08). Natural orifice specimen extraction was more frequent in the nonobesity group compared with the obesity group (28.2% versus 35.9%, *p* = 0.153), although this difference was not statistically significant.

**Table 2:** Post-matching of operative parameters among the obese and nonobese groups

	BMI ≥ 27 (*n* = 142)	BMI < 27 (*n* = 142)	*p*-Value
Operative time	312 (139)	280 (118)	0.287
Blood loss	50 (55)	50 (30)	0.07
< 100 ml	108 (76.1)	113 (79.6)	0.475
≥ 100 ml	34 (23.9)	29 (20.4)	
Diverting stoma			
Yes	72 (50.7)	64 (45.1)	0.342
No	70 (49.3)	78 (54.9)	
Conversion			
Yes	4 (2.8)	0	0.044*
No	138 (97.2)	142 (100)	
Anastomosis methods			
No	1 (0.7)	7 (4.9)	0.08
Hand sewn	22 (15.5)	25 (17.6)	
Staples	119 (83.8)	110 (77.5)	
Specimen extraction methods			
NOSE	40 (28.2)	51 (35.9)	0.153
Pfannenstiel incision	2 (1.4)	0	
Other abdominal incision	100 (70.4)	91 (64.1)	

*BMI* body mass index, *LapTME* laparoscopic total mesorectal excision, *TaTME* transanal total mesorectal excision, *NOSE* natural orifice specimen retraction

Table 3 presents postoperative results. Hospital length of stay and recovery milestones showed no significant variation between cohorts. Overall complication rates were notably elevated in the obesity group compared with the nonobesity group (40.1% versus 28.9%, *p* = 0.046). This increase was predominantly due to minor complications (Clavien–Dindo grade < 3), which occurred more frequently in the obesity group (35.9% versus 23.2%, *p* = 0.019), while major complication incidence (Clavien–Dindo grade ≥ 3) remained equivalent (4.2% versus 5.6%, *p* = 0.584). Common specific complications included ileus (8.5% versus 10.6%, *p* = 0.544), intraabdominal infection (IAI) (9.2% versus 7.0%, *p* = 0.514), and anastomosis leakage (4.2% versus 3.5%, *p* = 0.758), showing no significant differences. Surgical wound infection occurred more frequently in the obesity group (9.2% versus 1.4%, *p* = 0.004). Reoperation (*p* = 0.22) and permanent stoma rates (*p* = 0.157) were similar between the groups.

**Table 3:** Post-matching of short-term outcomes among the obese and nonobese groups

	BMI ≥ 27 (*n* = 142)	BMI < 27 (*n* = 142)	*p*-Value
Hospital stays	8 (5)	7 (5)	0.077
First flatus passage	2 (1)	2 (2)	0.361
First stool passage	2.5 (3)	3 (2)	0.2
Tolerated liquid diet	3 (3)	3 (3)	0.128
Tolerated soft diet	5 (4)	4 (4)	0.354
Remove Foley day	5 (4)	5 (3)	0.916
Overall complication			
Yes	57 (40.1)	41 (28.9)	0.046*
No	85 (59.9)	101 (71.1)	
Clavien–Dindo classification			
I	25 (17.6)	7 (4.9)	0.019*
II	26 (18.3)	26 (18.3)	
III	5 (3.5)	7 (4.9)	
IV	1 (0.7)	1 (0.7)	
V	0	0	
Major complication (Clavien–Dindo grade ≥ 3)	6 (4.2)	8 (5.6)	0.584
Minor complication (Clavien–Dindo grade < 3)	51 (35.9)	33 (23.2)	0.019*
Complication type			
Ileus	12 (8.5)	15 (10.6)	0.544
Anastomosis leak	6 (4.2)	5 (3.5)	0.758
IAI	13 (9.2)	10 (7)	0.514
Surgical wound infection	13 (9.2)	2 (1.4)	0.004*
Others	14 (9.9)	9 (6.3)	0.277
Reoperation			
Leakage	6 (4.2)	4 (2.8)	0.22
Bowel obstruction	0	2 (1.4)	
Others	0	2 (1.4)	
Permanent stoma	17 (12)	10 (7)	0.157

*BMI* body mass index, *LapTME* laparoscopic total mesorectal excision, *TaTME* transanal total mesorectal excision, *IAI* intraabdominal infection

### Risk factors for postoperative complications

Risk factor analysis for postoperative complications is detailed in Table 4. Regarding major complications, no statistically significant predictive factors were identified. For overall complications, obesity demonstrated a trend toward increased risk that bordered on statistical significance (OR 1.721, *p* = 0.05). Among the various operative techniques employed, none exhibited significant associations with overall complication rates. Additional variables including patient gender, tumor location, ASA score, pathological T-stage, N-stage, utilization of protective stomas, and preoperative adjuvant therapies demonstrated no meaningful correlations with either severe or total postoperative complications.

**Table 4:** Summary of the risk factors for postoperative complications

Major complications(Clavien–Dindo grade ≥ 3)			Overall complications		
	OR	*p*-Value		OR	*p*-Value
BMI > 27	0.668	0.507	BMI > 27	1.721	0.05*
Per 1 kg/m^2^ BMI increase	1.03	0.42	Per 1 kg/m^2^ BMI increase	1.06	0.41
LapTME	1		LapTME	1	
Robotic LAR	1.063	0.953	Robotic LAR	0.683	0.382
TaTME	0.967	0.139	TaTME	0.957	0.926
Age > 75 years	0.933	0.939	Age > 75 years	1.198	0.652
Male sex	1.613	0.474	Male sex	1.217	0.515
Upper rectum	1		Upper rectum	1	
Middle rectum	1.429	0.921	Middle rectum	1.226	0.687
Lower rectum	1.998	0.596	Lower rectum	0.845	0.724
ASA score			ASA score		
2	1		2	1	
3	4.423	0.069	3	1.328	0.341
pT-stage			pT-stage		
0 + 1 + 2	1		0 + 1 + 2	1	
3	1.252	0.746	3	1.199	0.551
4	4.115	0.209	4	1.602	0.430
pN-stage			pN-stage		
N0	1		0	1	
N1	0.761	0.717	1	0.536	0.072
N2	1.007	0.994	2	1.672	0.419
Diverting stoma	1.303	0.706	Diverting stoma	0.757	0.698
Neoadjuvant chemotherapy	0.226	0.121	Neoadjuvant chemotherapy	0.768	0.463
Neoadjuvant radiotherapy	1.095	0.913	Neoadjuvant radiotherapy	1.26	0.419

*BMI* body mass index, *LapTME* laparoscopic total mesorectal excision, *TaTME* transanal total mesorectal excision, *OR* odds ratio

### Histopathological parameters

Histopathological findings are presented in Table 5. Complete pathological response occurred in 5.6% of the obesity group compared with 4.2% in the nonobesity group (*p* = 0.584). Tumor type (*p* = 1) and differentiation (*p* = 0.671) showed no significant variation. Neither lymphovascular (21.8% versus 16.9%, *p* = 0.293) nor perineural invasion rates (16.9% versus 25%, *p* = 0.747) differed significantly between groups. Circumferential resection margin (CRM) positivity rates were equivalent across groups (4.2% versus 4.9%, *p = *0.776), while positive distal resection margin (DRM) rates also showed no difference (4.9% versus 7.0%, *p* = 0.453). Harvested lymph node counts were similar (23 ± 19 versus 24 ± 14, *p* = 0.6), and R1 resection showed no significant difference (9.9% versus 7.7%, *p* = 0.530).

**Table 5:** Post-matching pathological finding among the obese and nonobese groups

	BMI ≥ 27 (*n* = 142)	BMI < 27 (*n* = 142)	*p*-Value
pCR	8 (5.6)	6 (4.2)	0.584
Histology type			
Adenocarcinoma	132 (93)	132 (93)	1
Signet ring cell/mucinous	10 (7)	10 (7)	
Histology grade			
Grade I/II	121 (85.2)	125 (88)	0.671
Grade III	13 (9.2)	12 (8.5)	
Unclassified	8 (5.6)	5 (3.5)	
Lymphovascular invasion			
Positive	31 (21.8)	24 (16.9)	0.293
Negative	111 (78.2)	118 (83.1)	
Perineural invasion			
Positive	24 (16.9)	22 (25)	0.747
Negative	118 (83.1)	120 (72.5)	
CRM			
Positive	6 (4.2)	7 (4.9)	0.776
Negative	136 (95.8)	135 (95.1)	
Distal resection margin, length	1.5 (1.75)	1.4 (1.5)	0.158
Distal resection margin			
Positive	7 (4.9)	10 (7)	0.453
Negative	135 (95.1)	132 (93)	
Lymph node yield	23 (19)	24 (14)	0.6
R1 resection	14 (9.9)	11 (7.7)	0.530

*BMI* body mass index, *LapTME* laparoscopic total mesorectal excision, *TaTME* transanal total mesorectal excision, *pCR* pathological complete response, *CRM* circumferential resection margin

### Long-term outcomes

**Fig. 2:**
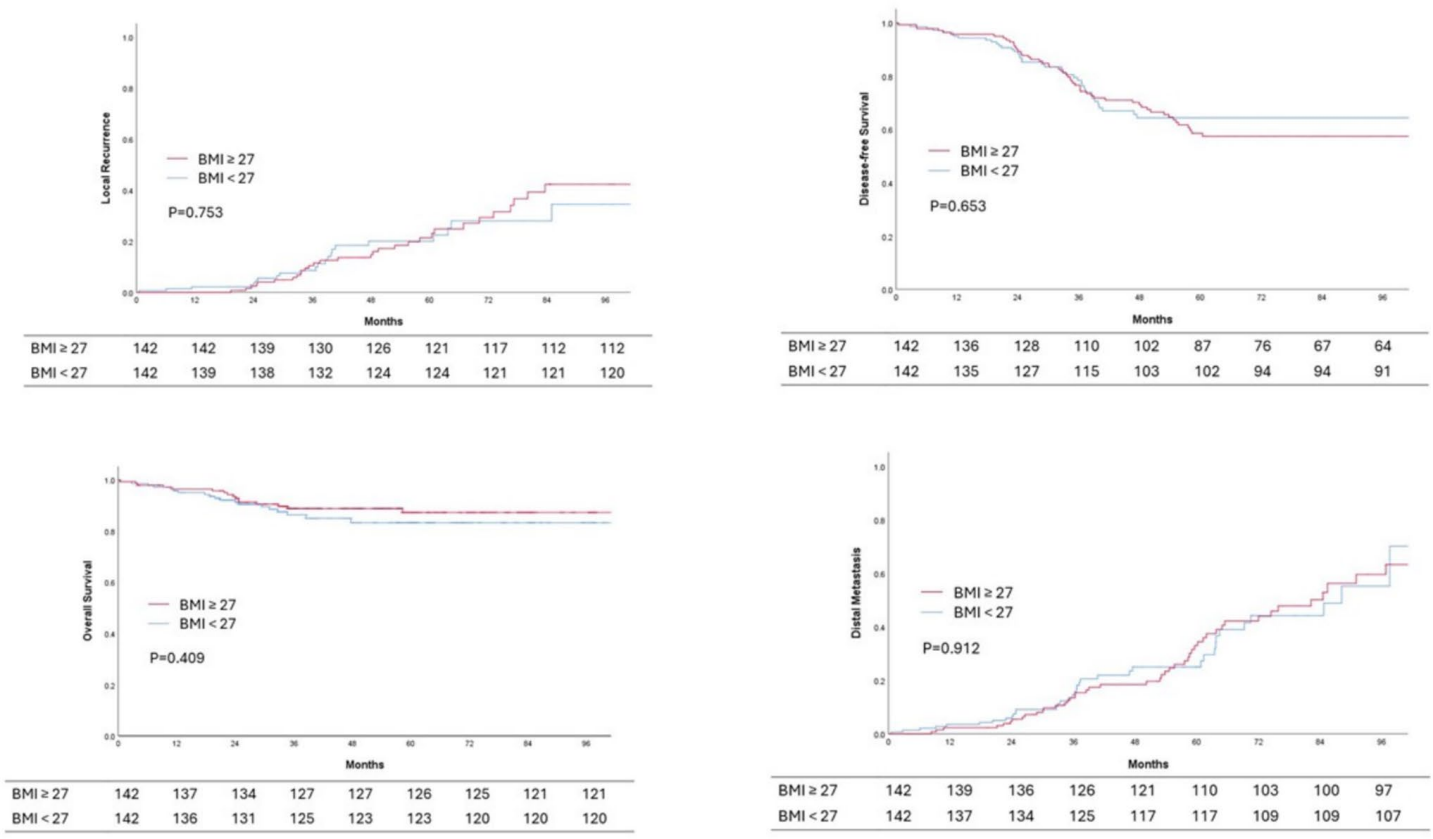
Kaplan–Meier survival curve

The median follow-up durations were 51.2 and 36.6 months for the obesity and nonobesity groups, respectively (*p* = 0.331). Figure 2 depicts the Kaplan‒Meier survival curve. LR rates were 15.5% and 23% (*p* = 0.753) and DM rates were 35.1% and 26.1% (*p* = 0.912), respectively. The 5-year OS rates (88% versus 89.4%, *p* = 0.409) and 5-year DFS rates (62.7% versus 72.5%, *p = *0.653) also did not reach statistical significance.

Additional exploratory subgroup analyses were performed to assess whether residual imbalances in tumor location and pT stage influenced long-term oncologic outcomes. Among patients with middle rectal cancer, no significant differences were observed between obese and nonobese groups with respect to LR, DM, OS, and DFS. Similarly, in the small subgroup of patients with pT4-stage tumors, long-term outcomes did not differ significantly between groups; however, these findings should be interpreted cautiously given the limited sample size. Accordingly, detailed subgroup tables were not presented.

## Discussion

The present study evaluated clinical and oncological results between elevated BMI group (above 27 kg/m^2^) and normal BMI groups following PSM in patients who underwent minimally invasive TME procedures at a high-volume tertiary center. After PSM adjustment, this analysis revealed comparable oncological outcomes between both patient groups. While obesity patients experienced increased conversion rate and overall postoperative complication rates, both short- and long-term outcomes remained equivalent across the groups.

With the global rise in obesity, the potential impact on postoperative outcomes following abdominal procedures has garnered considerable interest. In patients with obesity, surgical resection of rectal tumor presents distinctly greater technical complexity. Concerning operative parameters, conversion to open surgery remains to represent a major challenge in laparoscopic surgery. Previous meta-analyses showed significantly higher risk of conversion rate in obesity patients (OR 2.11−3.3), consistent with our findings [9, 15, 16]. Elevated conversion rates demonstrate the cumulative impact of anatomical and surgical challenges. Increased visceral adiposity and mesorectal fat accumulation compromise visualization within the confined pelvic space, particularly in male patients, obscuring tissue planes and increasing the potential for unintended damage to surrounding organs [17]. These factors may extend operative time and elevate intraoperative blood loss, both contributing to necessity of conversion [18]. Interestingly, as shown in previous studies, robotic surgery can overcome certain visualization and maneuverability constraints in particularly high-risk populations such as obese patients, thereby reducing conversion rates in subgroup analysis compared with LapTME (OR 0.22, 95% CI 0.07–0.71, *p* = 0.011; OR 0.46, 95% CI 0.21–0.99, *p * = 0.04) [19, 20]. Similarly, TaTME decreases conversion rates in obesity patients, as the transanal approach offers direct, magnified visualization of the lower rectum and mesorectal tissues, maintaining appropriate dissection planes even within a constricted, adipose-laden pelvis that compromises instrument maneuverability and field of view during LapTME procedures. TaTME decreases conversion rates in obesity patients as the transanal approach offers direct, magnified visualization of the lower rectum and mesorectal tissues, maintaining appropriate dissection planes even within a constricted, adipose-laden pelvis [21]. The restricted pelvic anatomy and proximity to surrounding structures during rectal surgery amplify procedure-specific complications. Surgeons must recognize these complexities during preoperative planning and consider patient selection carefully when considering minimally invasive approaches.

A prior investigation demonstrated that in patients with BMI above 27 kg/m^2^, more than 85% of Taiwanese, 66% of white, and 55% of Black patients exhibited at least one comorbidity such as hypertension, diabetes, hypertriglyceridemia, and hyperuricemia [4]. The chronic inflammatory processes and endothelial dysfunction linked to these obesity comorbidities may compromise wound healing capacity and elevate bleeding risk and hematoma formation [22]. Moreover, increased subcutaneous fat layers diminish vascular supply and oxygenation at surgical sites, limit antimicrobial drug distribution, and impair immune cell activity, thus creating an environment conducive to microbial growth and surgical site contamination, particularly in intestinal operations where native bacterial flora are abundant [23]. In our study, the obesity group had increased overall postoperative complication rates compared with the nonobesity group. The difference mainly resulted from surgical wound infection rate, aligned with previous studies, indicating that obesity represents an established risk factor for surgical wound infection, as seen in our results (*p* = 0.004) [9, 24, 25]. Additionally, an American College of Surgeons National Surgical Quality Improvement Program (ACS-NSQIP) study focused on proctectomy demonstrated progressive increases in superficial wound infections corresponding to elevated BMI classes [24]. Perioperative strategies require enhancement to mitigate the elevated risk. Body-weight-adjusted prophylactic antimicrobial dosing or broad-spectrum antibiotics to ensure sufficient tissue penetration, combined with standardized perioperative protocols, may effectively reduce infection rates.

Anastomotic leakage remains a critical complication following TME procedures. Anastomotic leakage is associated with increased morbidity and mortality, often leading to both surgical and radiological re-interventions. Moreover, it has been linked to higher rates of LR and lower long-term survival outcome [26]. We found no difference between the two groups in risk of anastomotic leakage in our study. An analysis from ACS-NSQIP found no significant association between BMI stratification and the 30-day risk of anastomotic leakage [27]. Notably, this result remained consistent after multivariate adjustment, supporting that under conditions of standardized perioperative care and high-quality TME in high-volume centers with stringent technical standards, obesity does not always translate to a higher rate of anastomotic leakage [27].

Current evidence indicates that long-term oncologic outcomes following TME are comparable between obesity and nonobesity patients. Several single-center studies have shown that the number of lymph nodes harvested, negative margin rates, and DFS rates are not diminished by elevated BMI, particularly when a high-quality mesorectal excision is achieved [10, 11]. For patients with mid-to-low rectal cancer, matched risk-adjusted analysis has demonstrated comparable 5-year OS and DFS rates among different BMI groups, suggesting that the technical difficulty and short-term complications associated with obesity do not translate into negative impact on long-term survival where oncologic principles and TME quality are maintained [28]. Evidence regarding pathological and specimen quality indicates that complete or nearly-complete TME and negative CRM rate are closely related to lower LR and DM rate [29]. The association is not affected by BMI, suggesting that the quality of surgical technique, rather than obesity itself, dictates the risk of recurrence.

Our study has several limitations. First, the single-center, retrospective design introduces a risk of selection bias and incomplete data capture. The retrospective analysis may not establish causality and is inherently limited by potential confounding variables. Second, although several covariates exhibited small residual imbalances after propensity score matching on the basis of their SMDs, we performed multiple additional analyses to evaluate the potential impact of these variables. Stratified and multivariable analyses focusing on the variables with the largest residual SMDs (gender, middle tumor location, and pT4-stage) consistently demonstrated that these factors did not modify the association between obesity and postoperative or oncologic outcomes. Importantly, even after these additional adjustments, obesity remained associated only with a higher conversion rate and an increased incidence of minor complications, while major complications and long-term oncologic outcomes remained unaffected. These findings support the robustness of our main conclusions despite the presence of minor residual imbalance. Additionally, BMI served as a practical surrogate for obesity classification but cannot precisely reflect visceral adiposity, and its accuracy may be compromised by extremes of height and gender variations in body fat distribution. Recently, visceral fat area (VFA), measured by CT imaging at specific abdominal cross-sections to assess intraabdominal adipose volume, serves as a valuable tool for investigating clinical concerns associated with central or visceral adiposity [30]. VFA serves as an increasingly important biomarker from a surgical perspective. It may be utilized to differentiate patients with colorectal cancer into more detailed stratification, enabling more precise perioperative assessments. In contrast, the Asian-defined cutoff of BMI ≥ 27 kg/m^2^ was adopted because metabolic abnormalities and visceral adiposity occur at lower BMI levels in Asian populations compared with Western populations. Although a BMI threshold of ≥ 30 kg/m^2^ is widely used internationally, the very small number of patients exceeding this level in our study, and generally in East Asian populations, substantially limits statistical power. Importantly, the BMI ≥ 27 kg/m^2^ cutoff more accurately reflects the distribution of obesity-related risk in Asian patients and better identifies individuals who may already present considerable intraoperative technical challenges despite not reaching a BMI of 30 kg/m^2^. Although the BMI cutoff of ≥ 27 kg/m^2^ was selected on the basis of established Asian-specific criteria and reflects the metabolic risk profile of East Asian populations, this threshold may limit the generalizability of our findings to Western populations, where obesity is more commonly defined using a BMI cutoff of ≥ 30 kg/m^2^. Therefore, caution is warranted when extrapolating our results to non-Asian cohorts with different body composition and obesity definitions.

## Conclusion

The retrospective study demonstrated that although obesity (BMI ≥ 27 kg/m^2^) was significantly associated with higher rates of conversion and minor postoperative complications, the short-term or long-term outcomes in patients who underwent minimally invasive TME approaches were comparable. The findings emphasize the need for tailored surgical and perioperative strategies for obesity patients. While intraoperative difficulties are more pronounced, structured postoperative care can help mitigate differences in complications.

## Supplementary Information

Below is the link to the electronic supplementary material.

Supplementary file1 (DOCX 21 KB)

## Data Availability

All datasets used or analyzed in this study are available from the corresponding author upon request.
