# Supplementary material for: Short- and long-term outcomes of minimally invasive total mesorectal excision in obese versus nonobese patients with rectal cancer: a propensity score matched study
Source: Tech Coloproctol. 2026 May 12;30(1):92. doi: 10.1007/s10151-026-03292-x (PMC13337685; doi:10.1007/s10151-026-03292-x)
Supplement: Supplementary file 1 — Supplementary file1 (DOCX 21 KB) [file 10151_2026_3292_MOESM1_ESM.docx]

**Table S1.** Post-matching of long-term outcomes among the obese and non-obese female patients with rectal cancer underwent restorative proctectomy

|  | | Obese females  (n = 42) | Non-obese females  (n = 56) | | p value | | | Obese Males  (n = 100) | | Non-obese Males  (n =86) | | p value |
| --- | --- | --- | --- | --- | --- | --- | --- | --- | --- | --- | --- | --- |
| Median follow up time (months) | 49.4 (35.8) | | | 34.6 (35.7) | | 0.428 | 39.4 (47.5) | | 37.1 (23.5) | | 0.518 | |
| Local recurrence | 12 (30.0) | | | 8 (14.3) | | 0.062 | 20 (20.0) | | 14 (16.3) | | 0.517 | |
| Median Time to LR (months) | 49.8 (43.2) | | | 43.9 (30.8) | | 0.768 | 22.3 (23.6) | | 27.2 (17.5) | | 0.459 | |
| Distant metastasis | 13 (33.3) | | | 17 (30.4) | | 0.759 | 34 (34.0) | | 24 (27.9) | | 0.256 | |
| Median Time to DM (months) | 46.8 (43.2) | | | 45.4 (41.1) | | 0.389 | 25.0 (20.2) | | 22.9 (41.7) | | 0.814 | |
| Deceased | 3 (7.1) | | | 6 (10.7) | | 0.545 | 18 (18.0) | | 16 (18.6) | | 0.915 | |
| 5–year OS | 95.2% | | | 92.9% | | 0.627 | 85% | | 87.2% | | 0.665 | |
| 5–year DFS | 64.3% | | | 71.4% | | 0.452 | 62% | | 73.3% | | 0.103 | |

LR: local recurrence; DM: distant metastasis; LapTME: laparoscopic total mesorectal excision; TaTME: transanal total mesorectal excisio
